# Antiproliferative Effects in Colorectal Cancer and Stabilisation in Cyclodextrins of the Phytoalexin Isorhapontigenin

**DOI:** 10.3390/biomedicines11113023

**Published:** 2023-11-10

**Authors:** Silvia Navarro-Orcajada, Francisco José Vidal-Sánchez, Irene Conesa, Francisco Escribano-Naharro, Adrián Matencio, José Manuel López-Nicolás

**Affiliations:** 1 Department of Biochemistry and Molecular Biology-A, Faculty of Biology, University of Murcia—Regional Campus of International Excellence “Campus Mare Nostrum”, E-30100 Murcia, Spain; 2 Department of Chemistry, University of Turin, Via P. Giuria 7, 10125 Turin, Italy

**Keywords:** isorhapontigenin, stilbene, cancer, encapsulation, solubility, stability

## Abstract

Isorhapontigenin has been proposed as a better alternative for oral administration than the famous resveratrol, as it shares many biological activities, but with a structure that could make its delivery easier. Although this hydrophobic structure could enhance bioavailability, it could also be a disadvantage in the development of products. In this research, we study the antiproliferative activity of this stilbene against colorectal cancer and overcome its limitations through molecular encapsulation in cyclodextrins. The cytotoxic activity against human colorectal cancer cells of isorhapontigenin was similar to that of resveratrol or piceatannol, supporting its use as a bioactive alternative. The study of the encapsulation through fluorescence spectroscopy and molecular docking revealed that the complexation satisfies a 1:1 stoichiometry and that HP-β-CD is the most suitable CD to encapsulate this stilbene. Through a spectrophotometric assay, it was observed that this CD could double the basal water solubility, exceeding the solubility of other hydroxylated stilbenes. The stability of these inclusion complexes was higher at a pH below 9 and refrigeration temperatures. Moreover, the use of CDs retained more than 78% of isorhapontigenin after storage for 12 weeks, compared to 15% in free form. Overall, these findings could help design novel formulations to better deliver isorhapontigenin.

## 1. Introduction

Isorhapontigenin (*trans*-3,4′,5-trihydroxy-3′-methoxystilbene) is a natural phytoalexin found in Asian medicinal plants (*Gnetum cleistostachyum*) and grapes [1]. It belongs to the stilbenoid family, along with resveratrol and piceatannol, from which it differs by the presence of a methoxy group in the 3′ position [2]. This slight difference in structure makes it not only more bioavailable than resveratrol, and therefore a better candidate for oral administration [3], but also more resistant to enzymatic oxidation because polyphenol oxidase or tyrosinase would no longer act on the 4′-OH. These facts encourage the use of isorhapontigenin instead of resveratrol or piceatannol to develop nutraceuticals.

In addition, isorhapontigenin is an active metabolite of piceatannol whose biotransformation is mediated by cathecol-O-methyl transferase (COMT) [4]. In rats, 17.70% of picetannol has been reported to be converted to this metabolite after intravenous administration.

For all these reasons, interest in the study of isorhapontigenin has considerably increased in the last ten years due to its potential benefits for human health. Recent publications that demonstrate its role as an antioxidant [5,6], anti-inflammatory [7], antimicrobial [8], cardioprotective [9,10], anticancer [11,12,13,14,15,16], antidiabetic [17] and neuroprotector [18,19] agent are proof of this. Although further comparative studies on the biological activity of this stilbene and its analogues are still needed to understand its great potential, a recent review highlights its biological activities and even calls it a successor to resveratrol [20].

However, the methoxy group of isorhapontigenin could worsen the poor water solubility of this family of bioactive compounds, preventing the development of stable formulations to deliver isorhapontigenin. Molecular encapsulation in cyclodextrins could improve the water solubility of this bioactive compound while protecting it from external agents. This type of complexation was effective with other hydroxylated stilbenes, such as resveratrol, piceatannol, oxyresveratrol, gnetol and pinosylvin [2]. However, the complexation of isorhapontigenin has not yet been studied and could increase the applications of this bioactive ingredient in biomedicine.

Cyclodextrins (CDs) are cyclic oligosaccharides obtained from the microbial degradation of starch. They consist of α-(1,4)-linked glucose units and can be classified as natural or modified. The most common are natural cyclodextrins with six (α-CD), seven (β-CD) and eight (γ-CD) glucose units, which are also approved as food additives (E-457, E-459 and E-458). Currently, modified cyclodextrins are used in the pharmaceutical industry, for example, 2-hydroxypropyl-β-CD (HP-β-CD) in the treatment of Niemann Pick type C disease.

Cyclodextrins possess an internal cavity of hydrophobic nature that allows them to form inclusion complexes with compatible molecules. Their external surface is mainly hydrophilic, which makes them highly water-soluble and able to increase the solubility of their guest molecules [21]. They can be used to remove undesired components, increase the extraction or production of bioactive compounds, design nanosensors, develop active packaging and carry bioactive compounds [22].

Considering the above, this research aims to determine and compare the anticancer activity of isorhapontigenin against colorectal cancer cells with the activity of other, most studied analogues, as well as analyse the molecular encapsulation of isorhapontigenin in natural and modified cyclodextrins by means of experimental and computational approaches in order to better deliver this bioactive compound. The influence of external factors of great interest for future applications, such as temperature and pH, is also considered and the thermodynamic parameters are established. Finally, the effect of this process on the water solubility and stability of isorhapontigenin is evaluated to validate the use of cyclodextrins in the formulations containing this stilbene.

## 2. Material and Methods

### 2.1. Materials

Natural cyclodextrins (α-CD, β-CD and γ-CD) were purchased from SigmaAldrich (Madrid, Spain). Methyl-β-cyclodextrin (M-β-CD, DS = 5.4) and 2-hydroxypropyl-β-cyclodextrin (HP-β-CD, DS = 5) were purchased from Carbosynth (Berkshire, UK). Isorhapontigenin was purchased from TCI (Belgium).

### 2.2. Equipment and Experimental Procedure

#### 2.2.1. Cell Line and Culture Conditions

Human colorectal cancer cell line Caco-2 was obtained from American Type Culture Collection (ATCC, Rockville, ML, USA) and was cultured in complete EMEM growth medium with 10% (*v*/*v*) foetal bovine serum, 2 mM L-glutamine and 0.1 mg/mL penicillin/streptomycin. Cells were grown according to ATCC guidelines in an incubator at 37 °C, 5% CO_2_ and 85% relative humidity.

#### 2.2.2. Cytotoxicity Test

Caco-2 cells (passage 34–37) were seeded in 96-well plates at 10,000 cells per cm^2^, filling the peripheral wells with sterile water to avoid evaporation effects. Cell concentration was determined with trypan blue using a TC10™ automated cell counter (Bio-Rad, Madrid, Spain). After incubation for 48 h, culture medium was replaced with fresh medium supplemented with 25, 50 and 100 µM of isorhapontigenin, resveratrol and piceatannol. All tested compounds were solubilised in dimethyl sulfoxide (DMSO 0.33% in the culture medium) and filter-sterilised (0.2 µm) before addition to the culture medium. Control cells (untreated) containing 0.33% DMSO were run in parallel and subjected to the same changes in medium. Each treatment was performed six times.

Cell proliferation was measured after 48 h incubation with the treatments through neutral red assay [23]. Briefly, a neutral red stock solution was prepared at 4 mg/mL in PBS and stored at room temperature in darkness. Treated cells were washed with PBS and incubated for 2 h with a working solution of neutral red (dilution 1:100 of the stock solution in complete growth medium). The cells were then washed again with PBS and air-dried, and an unstaining solution, consisting of a mixture of ethanol, water and acetic acid (1:1:0.02), was added. After 10 min with gentle oscillation, the absorbance was read at 540 nm on an FLUOstar Omega plate reader (BMG Labtech, Ortenberg, Germany). Background absorbance at 690 nm was subtracted from the measurement, as well as the absorbance of a blank with no cells. The relative cell viability of each treatment was determined through comparison to the control.

#### 2.2.3. Fluorimetric Assay

A Shimadzu RF-6000 spectrofluorometer equipped with thermostatically controlled cells was used to obtain the maximum excitation and emission wavelengths, setting bandwidths to 5 nm. In addition, a Kontron SFM-25 spectrofluorometer (Zurich, Switzerland) equipped with thermostatic cells with a xenon lamp source and 2 nm quartz cells was used to measure the relative fluorescence intensity, setting bandwidths to 2 nm.

The isorhapontigenin concentration was fixed to 25 μM (from an ethanolic solution at 3.87 mM) and the cyclodextrins set from 0 to 10 mM in 0.1 M sodium acetate buffer pH 3, 0.1 sodium phosphate buffer pH 5, 0.1 sodium phosphate buffer pH 7 and 0.1 sodium borate buffer pH 9. The solutions were incubated for thirty minutes at 5, 15, 25 or 35 °C.

#### 2.2.4. Determination of Stoichiometry and Encapsulation Constants

The Benesi–Hildebrand method [24] was used to calculate the stoichiometry and encapsulation constants for the inclusion complexes. According to this method, there are two potential models: a 1:1 model, in which one cyclodextrin molecule complexes one guest molecule (Equation (1)), and a 1:2 model, in which two cyclodextrins encapsulate one guest molecule (Equation (2)).

(1) Isorhapontigenin+CD ⇄ Isorhapontigenin−CD 

(2) Isorhapontigenin+2 CD ⇄ CD−Isorhapontigenin−CD 

The interaction between ligand and receptor can be quantified by the encapsulation constant (*K*_F_) (Equation (3)). Inclusion complexes are more stable if this parameter is higher.

(3) $$K_{F}=\frac{\left[Isorhapontigenin-{CD}_{x}\right]}{\left[Isorhapontigenin\right]\cdot {\left[CD\right]}^{x}}\;$$

where [*Inclusion complex*], [*Isorhapontigenin*] and [*CD*] are the equilibrium concentrations, and *x* is equal to 1 in 1:1 model and 2 in 1:2 model.

Measurement of the fluorescence signal in the absence and presence of cyclodextrins allows the calculation of the encapsulation constant using the following formula:

(4) $$\frac{1}{F-F_{0}}=\frac{1}{\left(F_{\infty }-F_{0}\right)\cdot K_{F}\cdot {\left[CD\right]}^{x}}+\frac{1}{F_{\infty }-F_{0}}\;$$

where *F*_0_ is the fluorescence intensity of isorhapontigenin in the absence of cyclodextrins, *F*_∞_ is the fluorescence intensity when all isorhapontigenin is complexed with cyclodextrins and *F* is the fluorescence intensity observed at each concentration of cyclodextrin. The linear plot of $\frac{1}{F-F_{0}}$ versus $\frac{1}{{\left[CD\right]}^{x}}$ gives $\frac{1}{\left(F_{\infty }-F_{0}\right)\cdot K_{F}}$ and $\frac{1}{F_{\infty }-F_{0}}$ as the slope and intercept, respectively.

#### 2.2.5. Determination of Thermodynamic Parameters

The enthalpy (Δ*H*°), entropy (Δ*S*°) and Gibbs free energy (Δ*G*°) of the most stable cyclodextrin complexes with isorhapontigenin were established using Equation (5).

(5) $$\ln K_{F}=\frac{-∆H{}^{\circ}}{RT}+\frac{∆S{}^{\circ}}{R}\;$$

where *T* is the temperature; *R* is the gas constant; and Δ*H*° and Δ*S*° are standard enthalpy and entropy changes of complex formation, respectively. The linear plot of $\ln K_{F}$ versus $\frac{1}{T}$ gives $\frac{-∆H{}^{\circ}}{R}$ and $\frac{∆S{}^{\circ}}{R}$ as the slope and intercept, respectively, and the Gibbs free energy change can be obtained from

(6) ∆G°=∆H°−T·∆S° 

#### 2.2.6. Molecular Docking

The isorhapontigenin molecule (CID 5318650) was obtained from the PubChem database (NCBI, USA), while natural cyclodextrins α-CD, β-CD and γ-CD were obtained from a crystal from the Protein Data Bank (PDB ID: 2XFY and 1Z0N) and the London South Bank University website. Modified cyclodextrins HP-β-CD and M-β-CD were built by adding hydroxypropyl or methyl groups to the β-CD. PRODRG (default parameters) was used to obtain the topology of modified cyclodextrins, while default topology was used for the remaining molecules. Autodock Tools (version 1.5.6) with default parameters and charges was used to generate the input files for docking. Autodock Vina [25] with default parameters was used to perform the molecular docking, setting a seed of 5000 and considering the flexible atoms of the cyclodextrins. Finally, PyMOL (Molecular Graphics System, version 1.3, Schrödinger, LLC) with the default parameters to display hydrogen bonds was used to obtain the graphical representations of the docking results.

#### 2.2.7. Water Solubility Measurement

The aqueous solubility of isorhapontigenin was determined in a Jasco V-630 Spectrophotometer using Thorlabs cuvettes CV10Q1400. First, the molar attenuation coefficient (ε) of isorhapontigenin was established for the first time by measuring the absorbance between 200 and 600 nm of increasing concentrations of isorhapontigenin in an ethanolic solution.

Next, saturated solutions of isorhapontigenin (1 mg/mL) in the absence and presence of different concentrations of HP-β-CD in water were incubated in the dark at room temperature for ten minutes. After centrifugation, the supernatant was diluted 1:100 in ethanol and the absorbance was read and quantified at 326 nm.

#### 2.2.8. Evaluation of the Stability of the Inclusion Complexes

Solutions of isorhapontigenin at physiological pH 7 with 25 μM of the free and encapsulated bioactive compound in low, medium and high doses of HP-β-CD (1, 5 and 10 mM, respectively) were stored at room temperature in the dark for 3 months. The remaining amount of isorhapontigenin was determined every two weeks by measuring the absorbance at 326 nm in a Jasco V-630 Spectrophotometer using Thorlabs cuvettes CV10Q1400. A blank was used with the buffer and ethanol at the same final concentration as the samples.

#### 2.2.9. Data Analysis

All experiments were conducted in triplicate. Regressions were performed using Sigma-Plot (version 10.0.0.54). One-way ANOVA test for independent measures was carried out for the cell proliferation experiments, while a *t*-test was performed for the encapsulation experiments using Rstudio (version 0.99.878) with a significance of *p* < 0.05.

## 3. Results and Discussion

### 3.1. Inhibition of Cell Proliferation in Human Colorectal Cancer with Isorhapontigenin and Its Analogues

**Figure 1:**
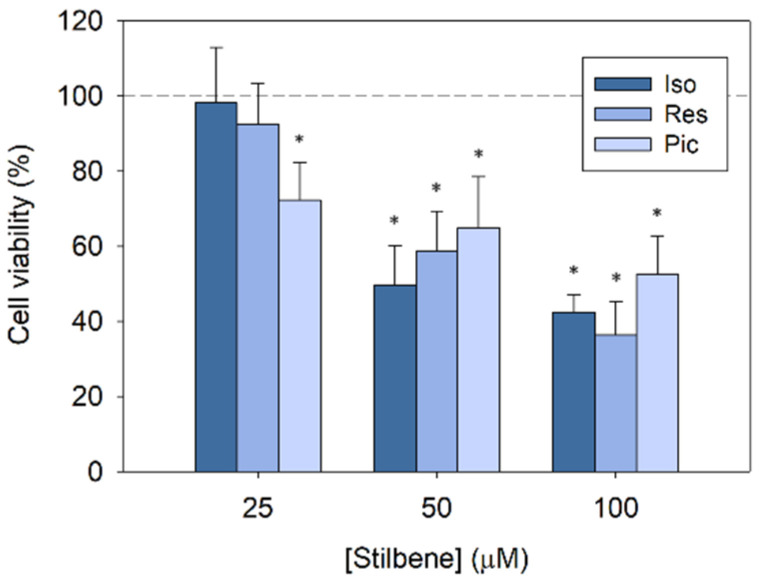
Cytotoxicity of isorhapontigenin, resveratrol and piceatannol on human colorectal cancer cell line (Caco-2) after 48 h treatment. * Significance *p* < 0.05.

The viability of Caco-2 cells after 48 h of incubation with isorhapontigenin and its analogues, resveratrol and piceatannol, decreased in a dose-dependent manner (Figure 1). Although, at 25 µM, only piceatannol showed cytotoxic activity, higher concentrations of stilbenes were able to achieve a remarkable inhibition of cancer cell proliferation, with 100 µM isorhapontigenin giving 43% cell viability. The survival of cells treated with isorhapontigenin at 50 µM was 50%, very similar to the viability obtained with resveratrol and piceatannol. Meanwhile, at the highest concentration (100 µM), there were no significant differences among stilbenes. Therefore, the slight modification of structure between isorhapontigenin and its more studied analogues, resveratrol and piceatannol, does not interfere with its ability to inhibit cell proliferation in colorectal cancer cells, supporting the use of isorhapontigenin as an alternative to these stilbenes.

Although there are no previous studies on the activity of isorhapontigenin against colorectal cancer, these results are in agreement with other authors that evaluated the cell growth inhibition in different cancer cells, such as prostate [16], lung [26], bladder [27] and breast cancer [14].

According to these studies, the SP1/EGFR signalling pathway is key to the anti-cancer effects observed following isorhapontigenin administration. Isorhapontigenin has been shown to inhibit the SP1 expression, transactivation and binding activity to the XIAP promoter, resulting in a down-regulation of XIAP (which is highly expressed in malignant cancer cells) [11]. It was also able to down-regulate cyclin D1 expression and induce G0-G1 cell cycle arrest through inhibition of SP1 [12,26,28]. In addition, isorhapontigenin has been reported to inhibit sphingosine kinase [14], induce the down-regulation of FOXO-1 [16] and decrease the expression of UCA1 [27]. These mechanisms could explain the antiproliferative effects observed in this work.

### 3.2. Determination of the Stoichiometry and Encapsulation Constants of Isorhapontigenin Complexes with Natural and Modified Cyclodextrins

**Figure 2:**
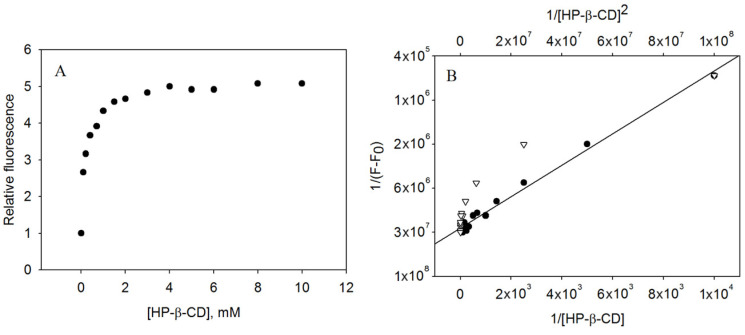
(**A**) Relative fluorescence of isorhapontigenin after encapsulation in HP-β-CD (25 °C pH 7). (**B**) Benesi–Hildebrand fitting of HP-β-CD complexation for (●) 1:1 model and (▽) 1:2 model.

The fluorescence of isorhapontigenin increased in a dose-dependent manner when CDs were added to the solutions, probably due to increased molecular stiffness (Figure 2A). Apart from γ-CD, all CDs were able to sufficiently increase the fluorescence signal to determine the stoichiometry and encapsulation constant of the inclusion complex (Table 1).

The Benesi–Hildebrand method fitted our experimental results quite well and revealed a 1:1 complexation model through a better correlation coefficient (R^2^) (Figure 2B), i.e., one CD molecule interacts with one isorhapontigenin molecule. This stoichiometry is in agreement with the encapsulation behaviour of other compounds of the stilbenoid family [2].

Among the natural CDs (Table 1), the β-CD (K_F_ = 3518.86 ± 175.94 M^−1^) complex was more stable than α-CD (K_F_ = 1265.55 ± 63.28 M^−1^) and γ-CD (K_F_ not determined). Meanwhile, modified CDs were able to improve the encapsulation efficiency of isorhapontigenin, as reflected by higher encapsulation constants than natural CDs (HP-β-CD, K_F_ = 6295.78 ± 314.79 M^−1^ and M-β-CD, K_F_ = 3823.47 ± 191.17 M^−1^). This order of preference of cyclodextrin was similar to that obtained with the analogue piceatannol in a previous study [29], although the isorhapontigenin constants were lower (piceatannol/HP-β-CD complex K_F_ = 14048 ± 702 M^−1^), probably because the hydroxyl group lost in isorhapontigenin decreased the interaction with cyclodextrin, i.e., by forming fewer hydrogen bonds, one of the most important interaction in this type of complexation.

As with piceatannol, the best CD for encapsulating isorhapontigenin was HP-β-CD, which reached the plateau at 3 mM (Figure 2A), indicating that above that concentration, all the bioactive compound forms a complex with the CDs.

### 3.3. Influence of pH on the Encapsulation Constants of Isorhapontigenin

**Figure 3:**
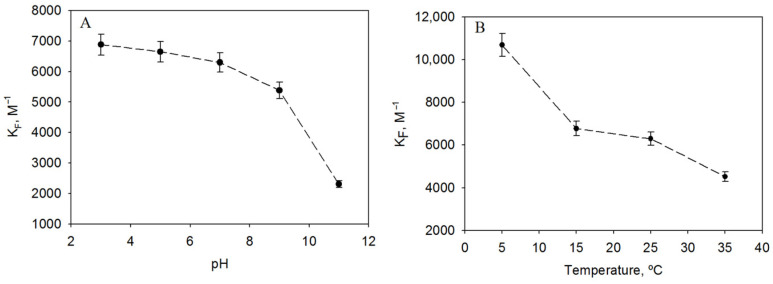
Variation in the encapsulation constants of isorhapontigenin complexes with HP-β-CD, with (**A**) pH and (**B**) temperature.

The pH of the medium seems to affect the stability of the inclusion complex with the best CD tested, HP-β-CD. At acidic and neutral pH, the variation in the encapsulation constants does not exceed 10% and K_F_ remains above 6000 M^−1^ (Figure 3A). However, above pH 7, the constants drop drastically, especially at pH 11 where K_F_ = 2306.30 ± 115.31 M^−1^ (a 63% drop compared to neutral pH).

Similar behaviour was previously observed with other stilbenes where the pH region in which the constant fell was related to a change in the protonation state of the molecule [2]. It seems that the fully protonated form of isorhapontigenin can form more stable complexes with CDs, probably due to the formation of hydrogen bonds.

### 3.4. Influence of Temperature on Encapsulation Constants of Isorhapontigenin

Since inclusion complexes can dissociate with increasing temperature and temperature can vary during the storage and delivery of commercial products, we determined the encapsulation constant at different temperatures. The isorhapontigenin complexes were more stable at lower temperatures (Figure 3B), giving a K_F_ = 10686.72 ± 534.34 M^−1^ under refrigerated conditions (5 °C). At 15 °C, 25 °C and 35 °C, the encapsulation constant decreased by 37%, 41% and 58%, reaching a K_F_ = 4520.16 ± 226.00 M^−1^ at the warmest temperature (35 °C).

The thermodynamic parameters were calculated according to Equations (5) and (6). The encapsulation reaction was exothermic with enthalpy ΔH° = −18.95 ± 0.95 kJ/mol, probably due to the displacement of water molecules from the cyclodextrin cavity to accommodate the guest molecule.

In contrast, the entropy value was positive (ΔS° = 8.53 ± 0.43 J/mol), which differs from previous results with other stilbenes, such as picetannol, gnetol or oxyresveratrol [2,29,30]. This was also observed in the complexation of other phenolic compounds in HP-β-CD, such as caffeic acid and rosmarinic acid. While the interaction with caffeic acid gave a negative entropy, the less polar characteristic of the latter resulted in a positive entropy [31]. Since isorhapontigenin is also less polar than other hydroxylated analogues, the release of structured hydration water in the bulk could explain the favourable entropy.

The process was spontaneous as the Gibbs free energy was negative (25 °C ΔG° = −21.49 ± 1.07 kJ/mol). The spontaneity of the reaction was close to that of piceatannol encapsulation (−23.5 ± 1.2 kJ/mol [29]).

### 3.5. Molecular Docking of Isorhapontigenin Inclusion Complexes with CDs

The computational results of isorhapontigenin complexation correlate well with the experimental results. The lowest score was obtained with HP-β-CD (Table 1), indicating that the encapsulation of isorhapontigenin on this CD was more efficient than with the other CDs. The HP-β-CD score was followed by β-CD, M-β-CD, α-CD and finally γ-CD.

**Figure 4:**
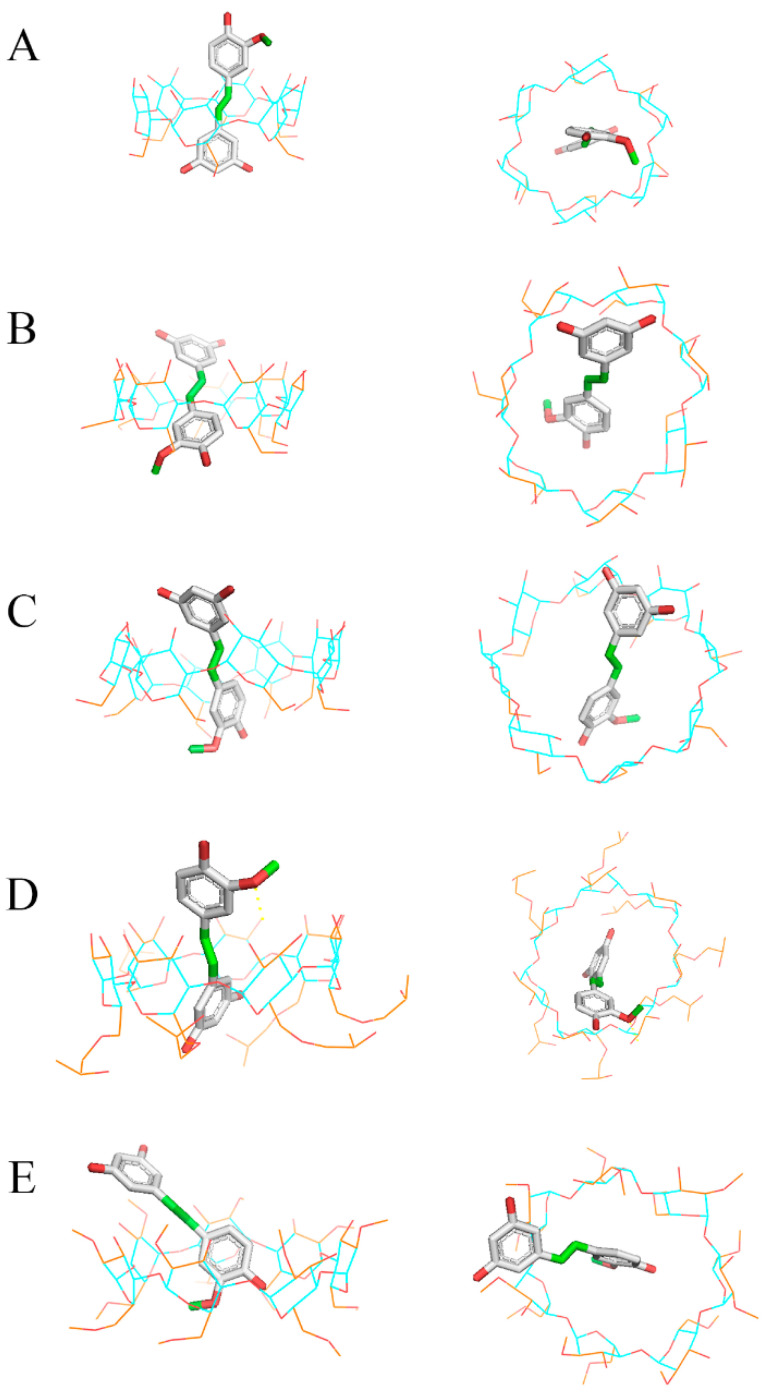
Molecular docking of isorhapontigenin with (**A**) α-CD, (**B**) β-CD, (**C**) γ-CD, (**D**) HP-β-CD and (**E**) M-β-CD. The flexible CD atoms are coloured orange and non-flexible atoms blue. Hydrogen bonds are yellow dotted lines.

In addition, the 3D configurations of each inclusion complex (Figure 4) revealed the formation of hydrogen bonds between the oxygen of the methoxy group of isorhapontigenin and a hydrogen from the secondary face of the HP-β-CD. Hydrogen bonds are one of the most relevant interactions that stabilise inclusion complexes, which revealed the best results with HP-β-CD.

### 3.6. Determination of the Improvement in Aqueous Solubility after Molecular Encapsulation

**Figure 5:**
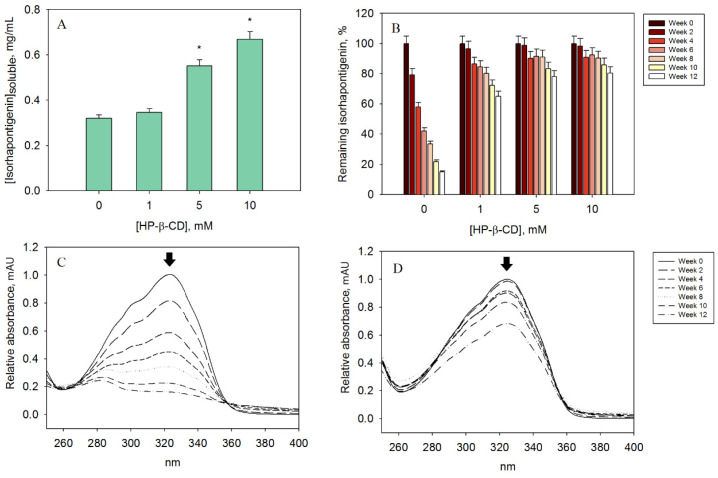
Improvement in the properties of isorhapontigenin after complexation with cyclodextrin. (**A**) Aqueous solubility at 25 °C, (**B**) stability at 25 °C pH 7, (**C**) stability spectrum of isorhapontigenin without cyclodextrins, (**D**) stability spectrum of isorhapontigenin with 5 mM of HP-β-CD. * Significance *p* < 0.05.

The aqueous solubility of isorhapontigenin in the absence of cyclodextrins was established at 0.32 mg/mL of water (ε_326_ = 39769 M^−1^cm^−1^), which is lower than the solubility of its hydroxylated analogue, piceatannol (0.50 mg/mL) [2]. Methoxylation of the 3′-hydroxyl group in piceatannol clearly decreased the aqueous solubility of stilbene, which could affect the development of nutraceuticals or aqueous-based drugs (Figure 5A).

Although the addition of a low concentration of HP-β-CD did not make a significant difference to this value, supplementation with 5 mM led to a 73% increase in solubility. The addition of 10 mM HP-β-CD doubled the basal solubility, reaching a solubility higher than the basal solubility of piceatannol. Therefore, cyclodextrin complexation could overcome potential solubilisation problems of this bioactive compound.

### 3.7. Time Course Evaluation of the Stability of the Inclusion Complexes

The results of the three-month storage of the inclusion complexes at room temperature showed the beneficial role of cyclodextrins in the potential applications of isorhapontigenin in the pharmaceutical, food or cosmetic industries. In the first six weeks, free isorhapontigenin was rapidly lost at a rate of approximately 10% of the initial amount per week (Figure 5B). After twelve weeks, the remaining isorhapontigenin without cyclodextrins was 15%, while the medium and high concentration of cyclodextrin still retained more than 78% of the stilbene, and the low concentration more than 65%.

The difference between 5 and 10 mM of HP-β-CD was not significant. Therefore, the use of the first concentration would be good enough to improve the stability of isorhapontigenin in formulations intended to be stored at room temperature.

It is observed that from the eighth week onwards, the spectrum without encapsulating agents flattened out, losing 326 nm as the maximum absorption peak at the tenth week (Figure 5C). This was not the case for the samples containing cyclodextrins, whose spectrum maximum was maintained throughout the experiment over time (Figure 5D).

## 4. Conclusions

Isorhapontigenin, a natural analogue of resveratrol and piceatannol, was found to have similar antiproliferative effects against human colorectal cancer cells, which encourages its use a bioactive alternative to these stilbenes. The physicochemical problems of this bioactive compound in the manufacture of stable formulations can be overcome through molecular encapsulation in cyclodextrins. This complexation followed a 1:1 model, in which one CD molecule interacts with one isorhapontigenin molecule. Natural and modified β-CDs gave higher encapsulation constants, with HP-β-CD being the best CD among them. The 3D modelling of the complexes revealed the formation of hydrogen bonds in the complexes with HP-β-CD. The inclusion complexes were more stable at lower pH values and temperature and were able to improve the water solubility and stability of isorhapontigenin after 3 months of storage, making possible the incorporation of this bioactive ingredient in water-based formulations. These results could be of great interest for the development of new nutraceuticals, drugs or cosmetics based on this stilbene.

## Figures and Tables

**Table 1:** Encapsulation constants according to Equation (4) and docking scores of the isorhapontigenin–cyclodextrin complexes (25 °C pH 7).

Cyclodextrin	R^2^		*K*_F_ (M^−1^)	Score
	1:1 Model	1:2 Model		
α-CD	0.932	0.719	1265.55 ± 63.28	−6.9
β-CD	0.709	0.467	3518.86 ± 175.94	−9.3
γ-CD	-	-	-	−6.6
HP-β-CD	0.992	0.925	6295.78 ± 314.79	−10.2
M-β-CD	0.942	0.744	3823.47 ± 191.17	−8.9

## Data Availability

No data was associated in the manuscript.
